# Association of colorectal cancer with genetic and epigenetic variation in *PEAR1*—A population-based cohort study

**DOI:** 10.1371/journal.pone.0266481

**Published:** 2022-04-07

**Authors:** Wen-Yi Yang, Benedetta Izzi, Adam P. Bress, Lutgarde Thijs, Lorena Citterio, Fang-Fei Wei, Erika Salvi, Simona Delli Carpini, Paolo Manunta, Daniele Cusi, Marc F. Hoylaerts, Aernout Luttun, Peter Verhamme, Sheetal Hardikar, Tim S. Nawrot, Jan A. Staessen, Zhen-Yu Zhang

**Affiliations:** 1 Department of Cardiology, Shanghai General Hospital, Shanghai Jiao Tong University School of Medicine, Shanghai, China; 2 Research Unit Hypertension and Cardiovascular Epidemiology, KU Leuven Department of Cardiovascular Sciences, University of Leuven, Leuven, Belgium; 3 Department of Epidemiology and Prevention, IRCCS NEUROMED, Pozzilli, Italy; 4 Department of Population Health Sciences, University of Utah, Salt Lake City, Utah, United States of America; 5 Division of Nephrology and Dialysis, IRCCS San Raffaele Scientific Institute, Milan, Italy; 6 Department of Cardiology, the First Affiliated Hospital of Sun Yat-Sen University, Guangzhou, Guangdong, China; 7 Department of Health Sciences, University of Milan, Milan, Italy; 8 School of Nephrology, University Vita-Salute San Raffaele, Milan, Italy; 9 Bio4Dreams SpA, Bresso, Milan, Italy; 10 Biomedical Science Group, University of Leuven, Leuven, Belgium; 11 Center for Molecular and Vascular Biology, KU Leuven Department of Cardiovascular Sciences, University of Leuven, Leuven, Belgium; 12 Huntsman Cancer Institute, University of Utah, Salt Lake City, Utah, United States of America; 13 Centre for Environmental Sciences, Hasselt University, Hasselt, Belgium; 14 Research Institute Association for the Promotion of Preventive Medicine, Mechelen, Belgium; University of Glasgow, UNITED KINGDOM

## Abstract

Platelet Endothelial Aggregation Receptor 1 (PEAR1) modulates angiogenesis and platelet contact-induced activation, which play a role in the pathogenesis of colorectal cancer. We therefore tested the association of incident colorectal cancer and genetic and epigenetic variability in *PEAR1* among 2532 randomly recruited participants enrolled in the family-based Flemish Study on Environment, Genes and Health Outcomes (51.2% women; mean age 44.8 years). All underwent genotyping of *rs12566888* located in intron 1 of the *PEAR1* gene; in 926 participants, methylation at 16 *CpG* sites in the *PEAR1* promoter was also assessed. Over 18.1 years (median), 49 colorectal cancers occurred, all in different pedigrees. While accounting for clustering of risk factors within families and adjusting for sex, age, body mass index, the total-to-HDL cholesterol ratio, serum creatinine, plasma glucose, smoking and drinking, use of antiplatelet and nonsteroidal anti-inflammatory drug, the hazard ratio of colorectal cancer contrasting minor-allele (*T*) carriers *vs*. major-allele (*GG*) homozygotes was 2.17 (95% confidence interval, 1.18–3.99; *P* = 0.013). Bootstrapped analyses, from which we randomly excluded from two to nine cancer cases, provided confirmatory results. In participants with methylation data, we applied partial least square discriminant analysis (PLS-DA) and identified two methylation sites associated with higher colorectal cancer risk and two with lower risk. In-silico analysis suggested that methylation of the *PEAR1* promoter at these four sites might affect binding of transcription factors p53, PAX5, and E2F-1, thereby modulating gene expression. In conclusion, our findings suggest that genetic and epigenetic variation in *PEAR1* modulates the risk of colorectal cancer in white Flemish. To what extent, environmental factors as exemplified by our methylation data, interact with genetic predisposition and modulate penetrance of colorectal cancer risk is unknown.

## Introduction

Platelets and endothelial cells abundantly express platelet endothelial aggregation receptor 1 (PEAR1), a transmembrane tyrosine kinase receptor. This protein mediates platelet contact-induced activation [1] and sustains platelet aggregation via activation of integrin αIIbβ3 [2, 3]. In both megakaryocytes and endothelial cells, knockdown of *PEAR1* downregulates expression of the phosphatase and tensin homologue (PTEN) [4, 5] and upregulates Akt phosphorylation. Both PTEN and Akt play a role in the proliferation of endothelial cells [5]. Silencing *PEAR1* in human endothelial cells results in increased tube formation, while complete *PEAR1* knockout in mice enhances neo-angiogenesis [5]. Tumor growth and metastasis rely on angiogenesis [6] and lymphangiogenesis, triggered by chemical signals emanating from tumour cells [7]. In mice, activated platelets are proinflammatory but blunt immunological responses [8], thereby shielding cancer cells from immunological recognition and facilitating the pathogenesis of colorectal cancer [9, 10]. In humans, the risk of colorectal cancer is lower in aspirin users [11].

Genetic variation in *PEAR1* modulates its expression [12, 13], platelet responses to agonists [14], and the interindividual variability in response to antiplatelet drugs [15, 16]. The *G* allele at *rs12041331* introduces a *CpG* site, increases methylation in the promotor of the gene, and enhances *PEAR1* expression [13]. We analysed the Flemish Study on Environment, Genes and Health Outcomes (FLEMENGHO) [17] to investigate the “*a priori*” hypothesis of a possible association of colorectal cancer with genetic variation in *PEAR1*, as captured by *rs12566888*, which is in complete linkage disequilibrium with *rs12041331*.

## Materials and methods

### Study design, recruitment and follow-up

FLEMENGHO complies with the Helsinki declaration for research in human subjects [18] and the protection of privacy in Belgium (www.gegevensbeschermingsautoriteit.be) and the European General Data Protection Rules (ec.europa.eu/info/law/law-topic/data-protection/eu-data-protection-rules_en). The Ethics Committee of the University Hospitals Leuven approved the study (Belgian registration number, B32220083510). Enrolment of FLEMENGHO participants started in 1985. From August 1985 until November 1990, a random sample of the households living in a geographically defined area of Northern Belgium was investigated with the goal to recruit an equal number of participants in each of six subgroups by sex and age (20–39, 40–59, and ≥60 years). All household members with a minimum age of 20 years were invited to take part, given that the quota of their sex-age group had not yet been satisfied. From April 1996 until May 2007 recruitment of families continued using the former participants (1985−1990) as index persons and including teenagers [17]. The initial participation rate was 78.0%. The participants were repeatedly followed up. In all study phases, the same standardised methods were applied to assess the clinical characteristics of participants, to administer questionnaires and to ascertain the incidence of adverse health outcomes. At each contact, participants gave or renewed informed written consent. Of 3343 participants, we excluded 811 from analysis, because blood stored in the biobank had been depleted or the DNA was degraded (n = 453), because participants were younger than 20 years old at enrolment and therefore not at risk (n = 393), because they had a history of colorectal cancer prior to enrolment (n = 4), because they had been lost to follow-up (n = 37), or because their *rs12566888* genotype was missing (n = 4). Thus, the number of participants included in the genetic analyses totalled 2532. Of those, 984 had DNA available for the epigenetic studies. Because of missing methylation information at one or more sites, we excluded a further 55 participants from the statistical analysis of the methylation data, leaving 929.

### Adjudication of cancer cases

The primary endpoint of the current study was incident colorectal cancer. Vital status of participants was assessed at annual intervals until 31 December 2016 via the Belgian Population Registry. The International Classification of Disease codes for the immediate and underlying causes of death were obtained from the Flemish Registry of Death Certificates. Information on the incidence of nonfatal cancer was collected via interview of participants at their homes or at the examination centre in the catchment area of the study, always using the same standardised questionnaire as at baseline (n = 2197) and in 335 participants via a structured telephone interview. Follow-up data were available from one visit in 698 participants, from two in 402, from three in 422, and from four or more in 675 participants. All colorectal cancer cases (ICD 8/9 153–154 and ICD 10 C18–C20) were adjudicated by consultation of the records of general practices, the four regional hospitals serving the study area, and the University Hospitals Leuven, the tertiary referral center for patients requiring highly specialised care.

### Genotyping and epigenetic measurement

**Fig 1:**
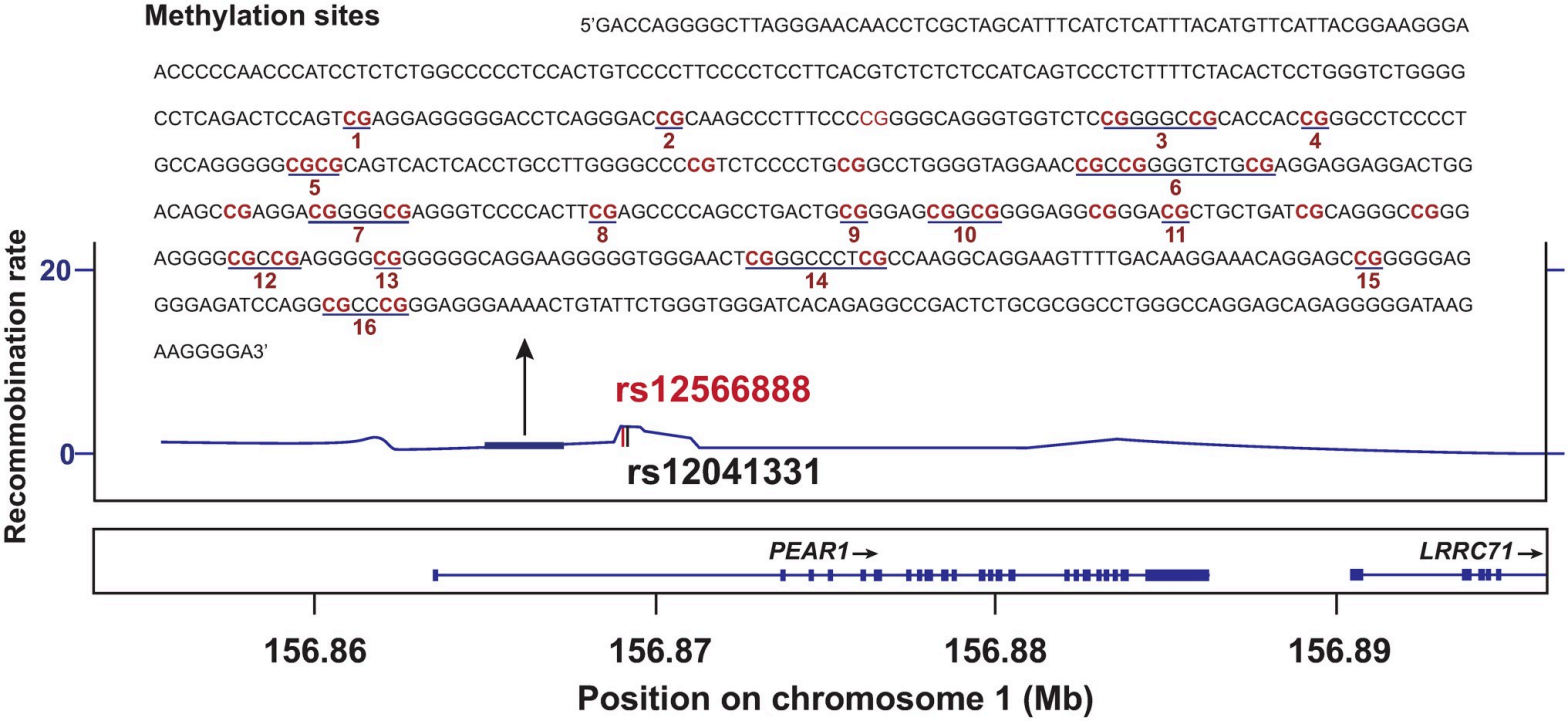
Plot of the *PEAR1* gene (1p13.1–12.3). The x-axis represents the physical position on chromosome 1 (build 37, hg19). The y-axis and the horizontal line indicate the recombination rate. SNPs *rs12566888* and *rs12041331* are in complete linkage disequilibrium (*r*^2^ = 0.99, D’ = 1.00). The 16 *CpG* sites in the studied DNA sequence of the promoter (bold lines) are consecutively numbered from 1 to 16 from the 5’ to 3’ gene terminals.

*PEAR1* (22,704 base pairs) maps to chromosome 1 (Fig 1). We genotyped *rs12566888* (*G*>*T*), which is in complete disequilibrium with *rs12041331*, the more commonly published polymorphism [12–16] (*r*^2^ = 0.99; D’ = 1.00) [19]. After extraction of genomic DNA from peripheral white blood cells [20], *rs12566888* was genotyped, using the TaqMan^®^ OpenArray^™^ Genotyping System (Life Technologies, Carlsbad, CA). DNA samples were loaded at 50 ng per μL and amplified on customised arrays. For analysis of the genotypes, we used autocalling methods implemented in the TaqMan Genotyper software version 1.3 (Life Technologies). Next, genotype clusters were evaluated manually with the sample call rate set above 0.90. Sixteen duplicate samples gave 100% reproducibility [17].

We quantified methylation at 22 *CpG* sites in the *PEAR1* promoter (Fig 1) on 1 μg of genomic DNA, using the Sequenom EpiTYPER MassARRAY platform (Agena Bioscience, Hamburg, Germany) [13] and the MethylDetector kit (Active Motif, La Hulpe, Belgium), according to the manufacturer’s instructions, except for the incubation period, which was lengthened to 16 hours. All PCR amplifications were performed in duplicate. To exclude possible inter-plate differences, a sample of K562 DNA, with known *PEAR1* methylation profile (around 90%), was carried through in each plate. The *PEAR1* amplicon was designed as described previously [13], using the Sequenom EpiDesigner software (http://www.epidesigner.com/), disregarding data when duplicate measurements had a SD of 5% or greater. Sequenom peaks with reference intensity above 2 and overlapping units were excluded from analysis, leaving 16 for statistical analysis.

### Replication analysis

We retrieved data from the publicly available Database of Genotypes and Phenotypes (dbGaP), including information from two studies conducted in the United States: the Diet and Lifestyle Study (DALS) [21, 22] and the Physician’s Health Study (PHS) [23], which are part of the Genetics and Epidemiology of Colorectal Cancer Consortium (GECCO; Accession number phs001078.v1.p1) [24]. Together, these two studies consisted of data on 1134 colorectal cancer cases and 1137 controls. For each of these studies, genomic DNA was extracted from blood samples or buccal cells by conventional methods, genotyped on Illumina platforms, and autosomal SNPs were imputed to a reference panel. The available dbGaP data did not include *rs12566888*. However, data were available on *rs12041331*, which is in complete linkage disequilibrium with *rs12566888* (*r*^2^ = 0·99; D’ = 1.00) [19].

### Statistical analysis

For database management and statistical analysis, we used SAS software, version 9.4 (SAS Institute, Cary, NC). For comparison of means and proportions within individuals, we applied a t-statistic and McNemar’s test and for comparisons between individuals, the large sample z-test or ANOVA and Fisher’s exact test, respectively. We tested Hardy-Weinberg equilibrium in unrelated founders, using the exact statistics available in the PROC ALLELE procedure.

First, we compared the cumulative incidence colorectal cancer between minor allele carriers (*T*) and major allele homozygotes (*GG*), using Cox proportional hazards regression adjusted for sex and age. Next, we applied multivariable-adjusted Cox regression to assess the colorectal cancer risk, while adjusting for sex, age, body mass index, the total-to-high density lipoprotein (HDL) serum cholesterol ratio, serum creatinine, plasma glucose, smoking and drinking, and use of antiplatelet drugs, including aspirin, non-steroidal anti-inflammatory agents, and dipyridamole. We checked the proportional hazard assumption by applying the Kolmogorov-type supremum test. We accounted for clustering within families as a random effect by using the SAS callable PROC SURVIVAL procedure as implemented in the SUDAAN 11.0.1 software (Research Triangle Institute, NC). To exclude a type-1 error, we bootstrapped the association analysis 1000 times, using a permutation test and by recalculating the hazard ratios after randomly excluding two up to nine cancer cases. For the replication analysis, logistic regression models were used to estimate odds ratios and 95% confidence intervals for the association between the risk of colorectal cancer and *rs12041331*.

For the epigenetic analysis, we first rank normalised distributions by sorting methylation measurements at each *CpG* site from the smallest to the largest and then applying the inverse cumulative normal function [25]. Next, to identify a set of independent latent factors that were linear combinations of the methylation values at 16 CpG sites, we applied partial least squares discriminant analysis (PLS-DA). This statistical approach allows constructing models for categorical outcomes in relation to highly correlated multidimensional predictors. We retained the smallest number of latent factors, for which the predicted residual sums of squares (PRESS) did not differ significantly (*p* > 0.10) from the model with the minimum PRESS value, as assessed by the van der Voet T^2^ statistic. The importance of each methylation sites in the construction of the PLS factors was assessed from the variable importance in projection (VIP) scores of Wold with the threshold set at 1.1.

In the in-silico analysis, we searched the *PEAR1* region under study for binding sites of transcription factors, using PROMO software (http://alggen.lsi.upc.es/cgi-bin/promo_v3/promo/promoinit.cgi?dirDB=TF_8.3) [26]. This software allows constructing weight matrices from known binding sites extracted from TRANSFAC (version 8.3, GeneXplain, Brauschweig, Germany) [27].

## Results

### Characteristics of participants

All 2532 participants were white Europeans, of whom 1297 (51.2%) were women. The study population consisted of 439 singletons, and 2094 related participants belonging to 53 single-generation families and 225 multi-generation pedigrees. At baseline (1985–2004), age averaged 44.8 years, body mass index 25.8 kg/m^2^, and total and HDL serum cholesterol 5.54 and 1.37 mmol/L, respectively. Among all participants, 44 (1.7%) had diabetes mellitus; 670 (26.5%) had hypertension; and 293 (11.6%) were taking aspirin (n = 133), nonsteroidal anti-inflammatory agents (n = 170), or dipyridamole (n = 8). Among 1297 women, 347 (26.8%) were smokers and 206 (15.9%) reported regular alcohol intake; among 1237 men these numbers were 421 (34.1%) and 506 (41.0%), respectively. In smokers, median tobacco use was 15 cigarettes per day (interquartile range, 10 to 20). In drinkers, the median alcohol consumption was 14 g per day (interquartile range, 8 to 25). Minor allele carriers (*T*) and major allele homozygotes (*GG*) had similar characteristics (0.10 ≤ *p* ≤ 0.94; Table 1).

**Table 1:** Characteristics of participants at enrolment by *rs12566888* genotype (1985–2007).

Characteristic	*T* allele carriers	*GG* homozygotes	All
Number	461	2071	2532
Number with characteristic (%)			
Women	247 (53.6%)	1050 (50.7%)	1297 (51.2%)
Current smoker	125 (27.1%)	643 (31.0%)	768 (30.3%)
Drinking alcohol	136 (28.9%)	591 (28.0%)	727 (28.1%)
Diabetes mellitus	4 (0.9%)	40 (1.9%)	44 (1.7%)
Hypertension	110 (23.6%)	561 (27.1%)	671 (26.5%)
Use of antiplatelet agents	54 (11.7%)	239 (11.5%)	293 (11.6%)
Mean of characteristic (SD)			
Age, years	44.4 (14.5)	44.8 (14.6)	44.8 (14.6)
Body mass index, kg/m^2^	25.8 (4.3)	25.8 (4.4)	25.8 (4.4)
Total cholesterol, mmol/L	5.51 (1.18)	5.55 (1.20)	5.54 (1.20)
HDL cholesterol, mmol/L	1.37 (0.41)	1.37 (0.38)	1.37 (0.38)
Total-to-HDL cholesterol ratio	4.39 (1.77)	4.37 (1.69)	4.38 (1.70)
Serum creatinine, μmol/L	90.7 (17.4)	92.0 (18.5)	91.7 (18.3)
Plasma glucose, mmol/L	5.13 (1.56)	5.04 (1.28)	5.06 (1.33)

HDL indicates high-density lipoprotein cholesterol. Antiplatelet agents included aspirin (n = 133), non-steroidal anti-inflammatory drugs (n = 170), and dipyridamole (n = 8). Diabetes mellitus was a fasting or random plasma glucose level of ≥ 7.0 mmol/L or ≥ 11.1 mmol/L (≥ 126 mg/dL or ≥ 200 mg/dL), or use of antidiabetic agents. Hypertension was a blood pressure of ≥ 140 mm Hg systolic or ≥ 90 mm Hg diastolic, or use of antihypertensive drugs. There were no differences between minor allele carriers and major allele homozygotes (0.10 ≤ *p* ≤ 0.94).

### Genetic analysis

In the whole population, the allele frequencies were 9.6% for *T* and 90.4% for *G*. The genotype frequencies were 0.9%, 17.3%, and 81.8% for the *TT*, *TG* and *GG* genotypes. Among 1125 unrelated founders, the corresponding allele and genotype frequencies were 9.8% and 90.2% and 1.2%, 17.0% and 81.7%, respectively. The allele frequencies complied with Hardy-Weinberg equilibrium (*p* = 0.26).

**Fig 2:**
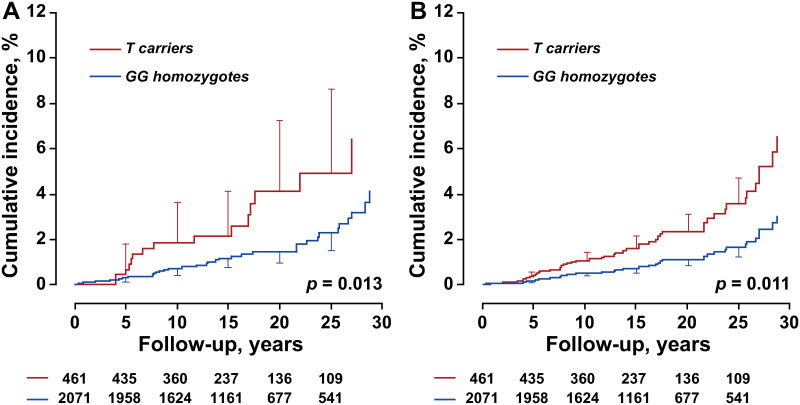
Unadjusted (A) and sex- and age-adjusted (B) cumulative incidence of colorectal cancer by *rs12566888* genotype. Vertical bars denote the standard error. Median follow-up was 18.1 years. *P* values are for the between-genotype differences. Tabulated data are the number of participants at risk by genotype at 5-year intervals.

Over a median follow-up of 18.1 years (5th to 95th percentile interval, 8.2 to 29.6 years), 49 patients were diagnosed with colorectal cancer. All incident colorectal cancer cases occurred in different pedigrees. Of these 49 patients, 17 died and 32 survived. With and without adjustment for sex and age, the cumulative incidence of colorectal cancer was similar in minor allele *TT* homozygotes and *TG* heterozygotes (*p* ≥ 0.92). *T* allele carriers were therefore pooled in subsequent analyses. The incidence of colorectal cancer was significantly higher (*p* ≤ 0.013) in minor allele *T* carriers than in *GG* homozygotes (Fig 2). While accounting for clustering of covariables within families and adjusting for baseline characteristics, including sex, age, body mass index, the total-to-HDL serum cholesterol ratio, serum creatinine, plasma glucose, smoking and drinking, and the use of antiplatelet agents, the hazard ratio associated with carrying the minor allele was 2.17 (95% confidence interval, 1.18 to 3.99; *p* = 0.013).

### Replication analyses

In the permutation analysis, the *T* allele carrier state was randomly assigned to 461 participants and *GG* homozygosity to the remainder of the cohort. A significance of less than 0.013 was reached in only 12 of 1000 runs. Randomly excluding from two to nine cancer cases kept significance in 92% of the runs, if two cases were excluded, and in 77% of the runs if nine cases were omitted from analysis. The mean hazard ratio over all bootstrap runs was 2.16 (95% confidence interval, 2.14 to 2.18).

In the 2271 dpGaP participants included in the replication analysis, the allele frequencies were 10.3% for *A* and 89.7% for *G* at *rs12041331*. The genotype frequencies were 1.5%, 17.7%, and 80.8% for the *AA*, *GA* and *GG* genotypes at this locus. Comparing with the *GG* homozygotes (n = 1835) at *rs12041331*, the odd ratio of the *A* allele carriers (n = 436) was 0.86 (95% confidence interval, 0.69 to 1.05, *p* = 0.15) without adjustment and 0.85 (0.69 to 1.05, *p* = 0.14) when adjusted for sex and age. The estimates were 0.80 (95% confidence interval, 0.63 to 1.01, *p* = 0.063) unadjusted and 0.79 (95% confidence interval, 0.62 to 1.01, *p* = 0.060) age-adjusted in men (n = 1644), and 1.07 (95% confidence interval, 0.70 to 1.64, *p* = 0.75) and 1.07 (95% confidence interval, 0.70 to 1.64, *p* = 0.75) in women (n = 627).

### Epigenetic studies

Table 2 compares the characteristics of the 929 participants included in the methylation study at enrolment and at the time when their methylation status was measured. Among these 929 participants, there were 16 colorectal cancer cases, diagnosed prior to (n = 11) or after (n = 5) the time point, at which methylation was assessed. The median interval (time of diagnosis minus time of methylation assessment) was -2.6 years (interquartile range, -4.4 to 1.9 years). For non-cases, the median time interval between censoring time minus methylation assessment was 3.1 years (interquartile range, -0.1 to 4.4 years). Methylation was not different according to the *rs12566888* genotypes (0.091 ≤ *p* ≤ 0.85) with the exception of higher methylation levels in *rs12566888 T* carriers compared with *GG* homozygotes at *CpG* sites 3 (*p* = 0.013), 4 (*p* = 0.029) and 6 (*p* = 0.048).

**Table 2:** Characteristics of 929 participants at baseline and at the time of the epigenetic measurements.

Characteristic	Baseline (1985–2004)	Epigenetic measurement (2005–2014)	Change (95% CI)
Number with characteristic (%)			
Women	465 (50.1%)	465 (50.1%)	…
Current smoker	237 (25.5%)	138 (14.9%)	–10.6 (–13.0 to 8.3)‡
Drinking alcohol	296 (31.9%)	364 (39.2%)	7.3 (4.1 to 10.5)‡
Diabetes mellitus	7 (0.8%)	57 (6.1%)	5.4 (3.9 to 6.8)‡
Hypertension	198 (21.3%)	502 (54.0%)	32.7 (29.5 to 36.0)‡
Use of antiplatelet agents	102 (11.0%)	236 (25.4%)	14.4 (11.2 to 17.7)‡
Mean of characteristic (SD)			
Age, years	42.4 (12.3)	56.9 (12.8)	14.5 (14.1 to 14.9)‡
Body mass index, kg/m^2^	25.6 (4.1)	27.2 (4.6)	1.7 (1.5 to 1.8)‡
Total cholesterol, mmol/L	5.40 (1.09)	5.08 (0.93)	–0.32 (–0.40 to –0.24)‡
HDL cholesterol, mmol/L	1.40 (0.38)	1.44 (0.37)	0.04 (0.03 to 0.06)‡
Total-to-HDL cholesterol ratio	4.14 (1.43)	3.70 (1.01)	–0.44 (–0.52 to –0.36)‡
Serum creatinine, μmol/L	89.1 (16.1)	88.4 (20.8)	–0.8 (–2.0 to 0.5)
Plasma glucose, mmol/L	5.05 (1.21)	4.94 (0.75)	–0.11 (–0.19 to 0.02)*

HDL indicates high-density lipoprotein cholesterol. Antiplatelet agents included aspirin (n = 151), non-steroidal anti-inflammatory drugs (n = 96), and at the follow-up examination also dipyridamole and clopidogrel (n = 12). Diabetes mellitus was a fasting or random plasma glucose level of ≥ 7.0 mmol/L or ≥ 11.1 mmol/L (≥ 126 mg/dL or ≥ 200 mg/dL), or use of antidiabetic agents. Hypertension was a blood pressure of ≥ 140 mm Hg systolic or ≥ 90 mm Hg diastolic or use of antihypertensive drugs. Changes are given with 95% confidence interval (in percent for categorical variables). Significance of the difference between baseline and follow-up:

* *p* ≤ 0.05;

^†^ *p* ≤ 0.01;

^‡^ *p ≤* 0.0001.

**Fig 3:**
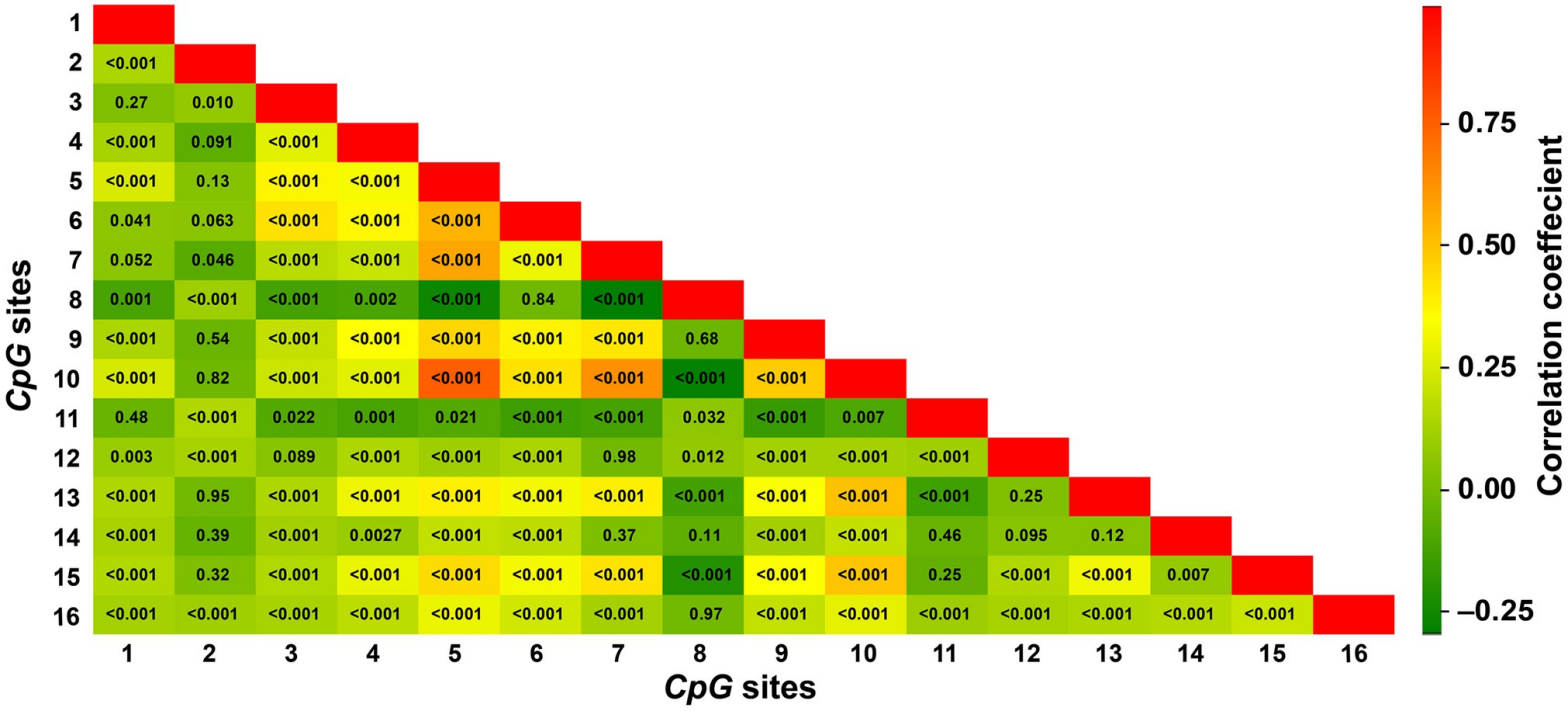
Correlation coefficients of the methylation level at 16 analysed *CpG* sites. The colour indicates the direction and magnitude of the correlation coefficients and the number within each box the significance.

**Fig 4:**
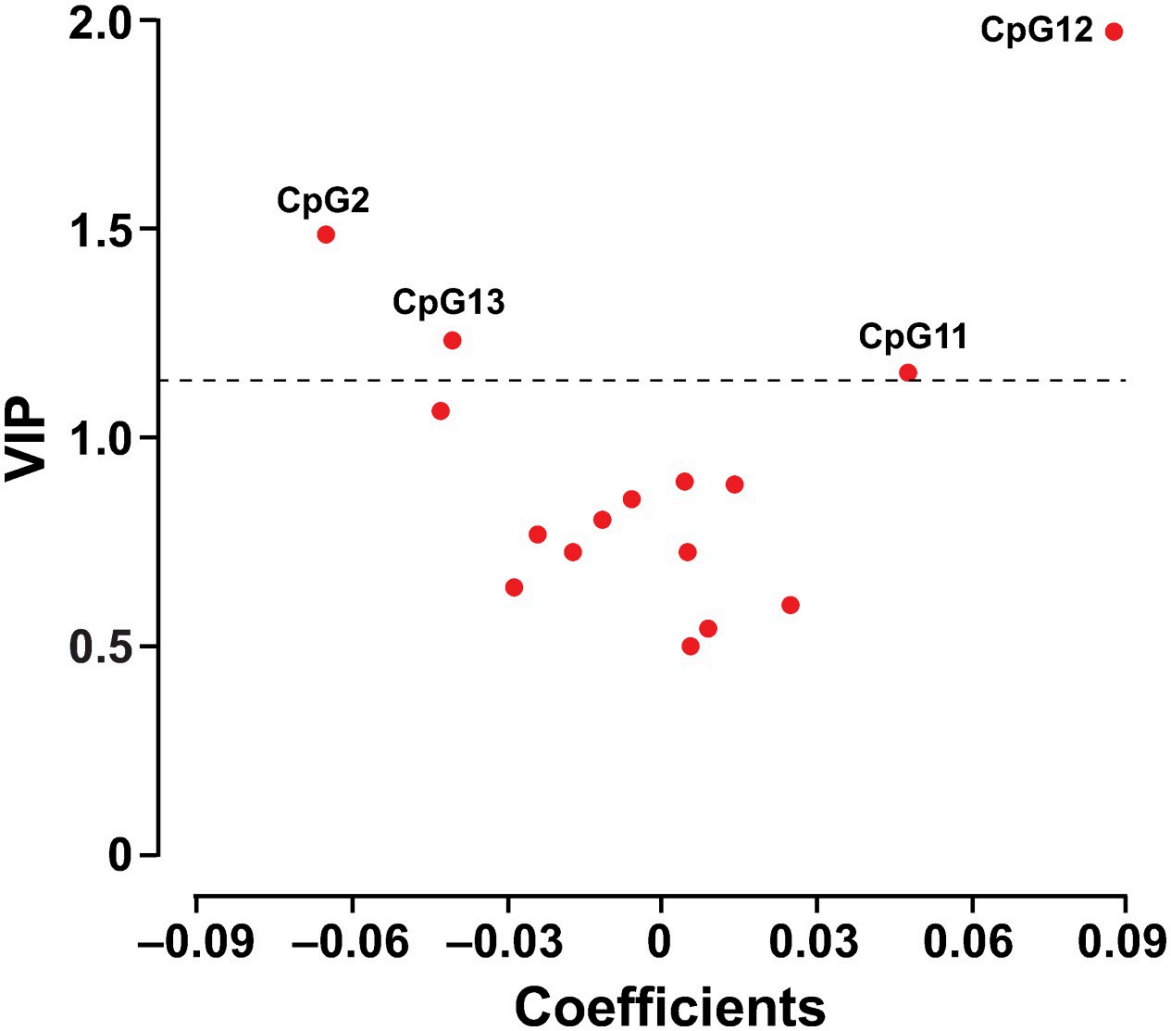
V-plots of variable importance in projection (VIP) scores against the centred and rescaled correlation coefficients generated by partial least-squares discriminant analysis. The VIP score indicates the importance of the methylation level at each site in the construction of the partial least-squares latent factors. The centred and rescaled correlation coefficients reflect the associations of colorectal cancer with the methylation level. The risk of colorectal cancer was standardised to the average (ratio or mean) in the whole study population of sex, age, body mass index, the ratio of total to high-density lipoprotein serum cholesterol, serum creatinine, smoking and drinking, the use of antiplatelet agents, and the time interval between the methylation measurement and diagnosis of colorectal cancer in cases or last follow-up in non-cases. Markers in the top left quadrant and top right quadrant of the V plot were respectively associated with lower and higher risk of colorectal cancer.

The methylation levels at the 16 *CpG* sites were highly correlated (Fig 3). The PLS-DA procedure identified two latent factors accounting for 24.6% and 6.7% of the variability in methylation at the 16 sites. Fig 4 shows the V-plot relating the colorectal cancer risk to the methylation levels at the 16 *CpG* sites. The risk of colorectal cancer was standardised to the average (ratio or mean) in the whole study population of sex, age, body mass index, the total-to-HDL serum cholesterol ratio, serum creatinine, smoking and drinking, the use of antiplatelet agents at the time of the methylation measurements, and the time interval between the methylation measurements and the diagnosis of colorectal cancer for cases or last follow-up for non-cases. Markers in the top left quadrant and top right quadrant of the V splot are respectively associated with lower and higher risk of colorectal cancer. Applying a VIP score of 1.1 showed that the methylation levels at *CpG* sites 2 and 13 were associated with lower risk and those at sites 11 and 12 with higher risk.

### In-silico analysis

Using the *PEAR1* methylation region as input, PROMO analysis identified transcription factors p53, PAX5 and E2F-1 as potentially binding to the *CpG* sites 2, 11, 12 or 13.

## Discussion

Our study confirmed our “*a priori”* hypothesis and to our knowledge is the first to relate in a randomly recruited population sample the risk of colorectal cancer to genetic and epigenetic variation in *PEAR1*. The genetic variation in *PEAR1*, as captured by *rs12566888*, is in complete linkage disequilibrium with *rs12041331*. The number of *G* alleles at *rs12041331* dose-dependently increased PEAR1 expression in platelets of 26 randomly selected participants enrolled in the GeneSTAR trial (Genetic Study of Aspirin Responsiveness) [12]. PEAR1 modulates angiogenesis [4, 5], which is required to sustain the growth of solid tumors [6]. The *G* allele at the *rs12041331* locus also introduces a *CpG* site into the first intron of the gene and influences binding of transcription factors [13]. In the gene promoter, we identified two *CpG* sites (2 and 13) associated with lower risk of colorectal cancer and two (11 and 12) with higher risk. The methylation status of the gene promoter regulates PEAR1 expression [13]. The in-silico analysis suggested that p53, PAX5 and E2F-1 are transcription factors potentially involved in the regulation of *PEAR1* expression. Further downstream, *PEAR1* expression stimulates *PTEN* expression [13], a direct inhibitor of colorectal carcinogenesis and an inhibitor of angiogenesis sustaining colorectal cancer growth [28]. Furthermore, PEAR1 stimulates megakaryopoiesis [13], and platelet activation [2, 3]. Activated platelets are central actors in orchestrating inflammatory reaction and vascular repair. The interaction with platelets facilitates cancer cells to escape from immune recognition [8, 9]. Our recent experimental data also showed that, via the methylation of its promoter, *PEAR1* mediates chromosome interactions with other genes, such as *PRCC* and *HDGF*, which are both involved in cell proliferation [29]. These observations highlight the potential involvement of genetic variation in *PEAR1* in modulating the risk of colorectal cancer.

**Fig 5:**
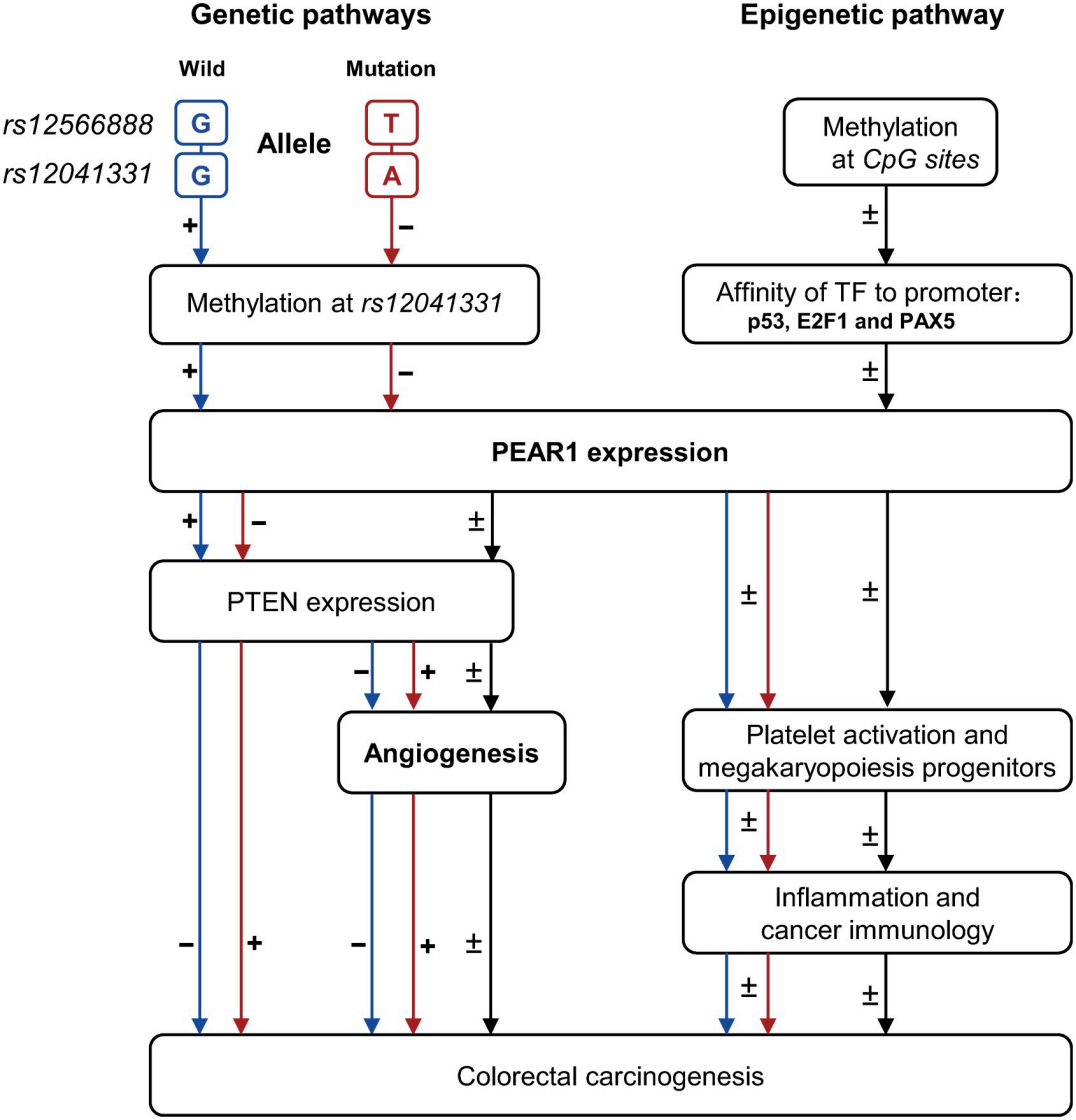
Potential pathways involved in the pathogenesis of colorectal cancer in relation to *rs12566888* and methylation status at *CpG* sites 2, 11, 12 and 13 in the *PEAR1* promoter. p53, PAX5, and E2F-1 are transcription factors identified in the in-silico analysis. *PTEN* represents phosphatase and tensin homologue.

The hypothetical pathways described above (Fig 5) are substantiated by an abundant literature on PTEN and p53 [30], PAX5 [31], and E2F-1 [32]. Genetic mutations in *PTEN* are associated with several cancer types, including colorectal malignancies [33–35]. The coding region of *PTEN* contains several repeat sequences, including two poly(A)6 tracts in exons 7 and 8. Mutations at these poly(A)6 tracts repeats are present in the cancer cells of approximately 18% of patients with colorectal cancer [33, 34]. Allelic losses in *PTEN* occur in some colorectal tumours [35]. Transcription factor p53 regulates a large number of diverse downstream genes, involved in the cell cycle. Dysregulation of p53 expression is one of the most frequent events contributing to the transformation of normal to colorectal cancer cells [30]. Among 995 patients undergoing surgery for colorectal cancer in the Sir Charles Gairdner Hospital, Nedlands, Australia, mutations in *p53* were found in 385 cases (39%) [36]. Among 13 colorectal cancer studies with a sample size greater than 50, the overall mutation frequency in *p53* was 806 (48%) among 1695 patients [36]. The paired box domain gene 5 (*PAX5*) encodes a transcription factor essential for B-cell differentiation [37]. In a study of the methylation profiles of 56 genes implicated in carcinogenesis, patients with colorectal cancer (n = 30) were compared with controls without lesions on colonoscopy (n = 30) [31]. *PAX5* methylation was observed in 26 (87%) patients with colorectal cancers and in 17 (57%) controls [31]. *PAX5* methylation as a biomarker to discriminate patients with colorectal cancer from healthy controls had a sensitivity of 87%, but a specificity of only 43% [31]. E2F-1 is a member of the E2F family of transcription factors and plays a key role in the cell cycle by binding with a retinoblastoma tumour suppressor protein (Rb) [38]. The control of the Rb/E2F pathway is disrupted in all human cancers [39], including colorectal cancer. Finally, a growing body of evidences supports that the methylation phenotype of *CpG* sites is one of underlying mechanisms in colorectal carcinogenesis [40], especially during the early stages of oncogenic transformation [41–43].

Of 3395 publications available in the genome-wide association study database of the National Human Genome Research Institute (www.ebi.ac.uk/gwas/search), at the time of writing this article (2021), 51 dealt with colorectal cancer. These studies identified 639 SNPs associated with colorectal cancer with a *p* value of less than or equal to 1 × 10–5. *PEAR1* SNPs did not appear in the list. However, given the adjustment of significance for multiple testing, genome-wide association studies are unlikely to detect an association between an adverse health outcome and a genetic variant, if the frequency of the risk carrying allele is low, as is the case for the minor *T* allele at *rs12566888* (9.6%). Permutation analyses within the FLEMENGHO cohort supported the association colorectal cancer with *PEAR1* gene variants. The external validation only showed weak association in male dbGaP participants. Our prospective study tested an “*a priori*” hypothesis and met several Bradford-Hill criteria to infer causality: (i) the consistency of the association across genetic, epigenetic and in-silico analyses; (ii) temporality, genetic variability preceding colorectal carcinogenesis; (iii) plausibility based on the aforementioned experimental studies [2–4, 13] and the observation that there is an inverse association between the risk of colorectal cancer and the use of aspirin [11]; and (iv) analogy observed with genetic variability in *PTEN* [33–35], which is directly regulated by PEAR1 [4].

Our study must also be interpreted in the context of its limitations. First, our current findings in white Flemish are difficult to extrapolate to other ethnicities [44]. Second, we showed previously that methylation of the promoter regulates *PEAR1* expression [13], but in view of Belgian privacy regulations, currently tightened by the European General Data Protection Regulation (https://ec.europa.eu/info/law/law-topic/data-protection/eu-data-protection-rules_en), we were not granted access to the biopsies from the cancer cases included in our current study. Third, as in all observational studies, we cannot exclude residual confounding by unmeasured risk factors. Fourth, methylation of the *PEAR1* promoter was assessed 12.5 years after collection of the baseline data. However, the methylation of the *CpG* sites was not affected by advancing age and our analyses were adjusted for age. Finally, we measured methylation of DNA samples extracted from peripheral white blood cell instead of colorectal cancer cells. However, methylation profiles of oncogenic or cancer suppressor genes harvested from white blood cell [45], plasma [46], or serum [47] are likely to be concordant with the profiles in cancer cells.

In conclusion, starting from the concept that growth of solid tumours requires angiogenesis to which PEAR1 contributes, this study demonstrated in a representative population sample that the risk of colorectal cancer was associated with genetic variation in *PEAR1* and methylation of the *PEAR1* promoter. DNA methylation at each *CpG* site may influence binding of transcription factors, thereby directly controlling gene expression [48] or regulating chromosome interactions with distant genomic regions [49]. Both mechanisms are relevant to *PEAR1* methylation (Fig 5). However, given that the genome-wide association studies on colorectal cancer did not include *rs12566888* and the weak association in the replication cohort, further epidemiological data and molecular and animal experiments are warranted for resolving the disparities.
